# Retrospective analysis of antimicrobial resistance of *Salmonella* spp. isolated from livestock and its environment in Thailand

**DOI:** 10.3389/fvets.2025.1584940

**Published:** 2025-06-09

**Authors:** Preeda Lertwatcharasarakul, Sakuna Phatthanakunanan, Phitsanu Tulayakul

**Affiliations:** ^1^Department of Veterinary Pathology, Faculty of Veterinary Medicine, Kasetsart University, Nakhon Pathom, Thailand; ^2^Faculty of Veterinary Medicine, Kamphaeng Saen Veterinary Diagnostic Center, Kasetsart University, Nakhon Pathom, Thailand; ^3^Department of Veterinary Public Health, Faculty of Veterinary Medicine, Kasetsart University, Nakhon Pathom, Thailand

**Keywords:** *Salmonella*, antimicrobial resistance, multidrug resistance, environment, livestock

## Abstract

**Introduction:**

A retrospective study of non-typhoidal *Salmonella* isolation from poultry and pig farms in Nakhon Pathom and Suphan Buri provinces was conducted from 2008 to 2015. The aim of study was to examine the prevalence of antimicrobial resistance and class I integrons related to gene resistance of *Salmonella* in livestock and its environment.

**Methods:**

A total of 636 *Salmonella* isolates was collected from livestock and environmental samples. The isolates included 1.42% *S.* Typhimurium, 4.40% *S. Enteritidis*, and 1.26% *S. Virchow*; however, neither *S. Infantis* nor *S. Hadar* were found. All *Salmonella* isolates was tested for antimicrobial susceptibility and minimum inhibitory concentrations (CLSI Vet03-S2 2014, NCCLS standard).

**Results:**

The top three drug resistances were to cephalexin, gentamicin, and amoxicillin. *S.* Typhimurium showed resistance rates of 100%, 100%, and 22.22% to these antibiotics, respectively; *S. Enteritidis* showed resistance rates of 100%, 100%, and 90.91%; and *S. Virchow* revealed resistance at the rates of 50%, 50%, 12.50%, respectively. The conserved segment integrase 1 and gene cassette were found by polymerase chain reaction (PCR) in all serotypes. The resistance gene of *aad*b, *Int*I1, *aac*(6′)-la, *aac*(6′)-lb, *bla*_PSE-1_, *Cml*A, *Sul*, *dfr*A1, A10 and A12 were not detected from *S.* Typhimurium and fewer resistance genes were detected when compared to the other two subtypes.

**Discussion:**

These findings could be used to set up the prevention and control strategies for addressing future antimicrobial resistance of *Salmonella*, which remains a major food safety concern.

## Introduction

*Salmonella* spp. is a Gram-negative, non-spore-forming bacterium in the *Enterobacteriaceae* family. It is facultative anaerobe, digests glucose, generates hydrogen sulfide, but cannot ferment lactose. *Salmonella* is a mesophilic bacterium and grows well between 35–42°C, however, the lowest temperature could be growth at 5.2°C and it could be survived within a pH range of 4.5–9.0 (1). *Salmonella* spp. is a significant bacterium in veterinary public health because it causes Salmonellosis, a serious gastrointestinal disease that affects populations worldwide, including Thailand and South Asia (2, 3). It causes of foodborne illness worldwide and global public health impact. In case that the multidrug resistance (MDR) happened in *Salmonella*, it has been exacerbating with the limitation of disease treatment options (4). As indicated in the “Thailand Disease Outbreak Laws 2015,” *Salmonella* is categorized as an outbreak livestock disease affecting both humans and animals. The most common serovar causing human salmonellosis in Thailand was *Salmonella Enterica Weltevleden*, the data from 1993 until 2002 (5). Non-host specific serovars such as *Salmonella Enteritidis* (*S. enteritidis*)*, S.* Typhimurium, *S.* Hadar, *S. infantis*, and *S.* Virchow cause non-typhoidal *Salmonella* (NTS) diseases in animals, including cows, buffaloes, pigs, ducks, chickens and Tilapia fish (6). Infected animals may be asymptomatic carriers, transmitting the disease through food products to consumers. Infected patients may experience gastrointestinal tract infections and, in some cases, bacteremia, particularly in children, senior adults, or immunocompromised individuals, alongside antimicrobial resistance (7). Currently, antibiotic resistance in meats and animal products is an escalating problem impacting human health due to the extensive use of antibiotics in livestock for disease control, prevention, treatment, and growth promoters. Reports indicate antimicrobial resistance in swine farms to antibiotics such as Ampicillin, Tetracycline, Streptomycin, Sulfamethoxazole plus Trimethoprim, and Chloramphenicol especially for *tet*A of tetracycline resistance isolates (8), and similar resistance patterns have been observed in poultry farms (9, 10). *Salmonella* isolates from Tilapia showed resistance rates of 5.5% for Ceftazidime, Chloramphenicol, Meropenem, Nitrofurantoin and Streptomycin and 22.2% to Penicillin-G in the fish sample (6). There was also reported of MDR from 3 of *Salmonella* spp. and 2 of *E coli* isolated from this study.

The antimicrobial resistance of *Salmonella* spp. is increasing, leading to multidrug resistance. Resistance genes can be transferred to other bacteria via important resistance mechanisms, notably through class 1 integrons, which contain multiple resistance genes in the form of gene cassettes. These integrons, housing over 100 antimicrobial resistance genes (11), result in multidrug resistance patterns in *Salmonella*. Additionally, mobile genetic elements facilitate the transfer of resistance gene cassettes between similar and different bacterial species. The use of antimicrobials in livestock can thus lead to outbreaks of multidrug-resistant genes. As Thailand exports significant quantities of livestock products, stringent disease control and prevention measures, particularly against *Salmonella* spp., are necessary (7, 12). The purpose of this study is to conduct a retrospective analysis of antimicrobial resistance patterns of *Salmonella* spp. and their functions on class 1 integrons from samples collected from livestock. This study will provide valuable data to mitigate the economic and public health impact of *Salmonella* outbreaks in Thailand.

## Materials and methods

### Sample collection

The retrospective study utilized *Salmonella* isolation data collected from livestock and their environments in the Nakhon Pathom and Suphan Buri provinces in the western region of Thailand from 2008 to 2015. Samples were collected from six swine farms and seven poultry farms (six duck farms and one broiler farm), with the total of 630 isolated samples. Bacterial isolation was performed using cloacal swabs, soil, and water samples collected from the farms. Among these, 86 samples were from cleaning water before and after use, 39 samples from piglets’ floors, 24 samples from piglets, 18 soil samples, and 4 samples from drinking water collected before and after treatment. Additionally, 544 cloacal swabs were collected from poultry farms, including 523 samples from duck farms and 21 samples from the broiler farm.

### Bacterial isolation and identification

*Salmonella* was isolated using the standard methods outlined in ISO 6579:2002, which identifies non-host-specific serovars such as *S.* Typhimurium, *S. enteritidis*, *S*. Virchow, *S. infantis* and *S.* Hadar. The collected samples were initially placed in lactose broth and incubated at 37°C for 18–24 h. They were then transferred to Tetrathionate broth and Rappaport Vassiliadis medium (RVS), followed by plating on Xylose-lysine Deoxycholate agar (XLD) and Brilliant Green Phenol Red Lactose Sucrose agar (BPLS) and incubated at 37°C for 18–24 h. Subsequently, isolated colonies of specific *Salmonella* were selected and tested on Triple Sugar Iron agar (TSI), subjected to the Urease test and L-lysine decarboxylase test at 37°C for 18–24 h, and then examined with a Slide Agglutination test using poly OH antigen for *Salmonella* spp. serovars identification. The purified *Salmonella* colonies were stored in 1.5 mL of skim milk at −20°C until further analysis.

### Antibiotic sensitivity testing

The antibiotic sensitivity testing for *Salmonella* spp. was conducted by determining the Minimum inhibitory concentration (MIC) using a VITEK II machine, operated according to the CLSI Vet03-S2 2014 NCCLS standard (The National Committee for Clinical Laboratory Standards). This testing included 16 antibiotics: Amikacin, Amoxicillin/Clavulanic Acid, Ampicillin, Cefalexin, Cefovecin, Cefpodoxime, Ceftiofur, Chloramphenicol, Enrofloxacin, Gentamicin, Imipenem, Marbofloxacin, Nitrofurantoin, Piperacillin, Polymyxin B, Rifampicin, Tetracycline, Tobramycin, and Trimethoprim/Sulfamethoxazole. The frequency comparison was tested using Fisher’s exact test with the statistically significant difference at (*p* < 0.05) by GraphPad Prism Statistical Software, version 5.01, 2007 (GraphPad Software, Institute., USA.).

### The study of genotypic resistance gene of *Salmonella* spp.

The study of class I Integron by the integrase gene, *Int*I1, and gene cassettes located at the conserved segments of 5’CS and 3’CS was conducted using Polymerase Chain Reaction (PCR), referenced in Table 1. The Ampicillin-resistance genes are *Bla*psc1 (n = 5) and *Bla*TEM (n = 5); Chloramphenicol-resistance genes are *cat*A (n = 5), *cat*B (n = 5), and *cml*A (n = 5); Tetracycline-resistance genes are *tet*A (n = 5) and *tet*B (n = 5); Trimethoprim-resistance genes are *dfr*A1 (n = 5), *dfr*A10 (n = 5), and *dfr*A12 (n = 5); and the Sulfamethoxazole-resistance gene is *sul*1 (n = 5).

**Table 1:** Demonstrate of primers using in this study.

Target gene or region	Primer	Sequence of primer (5′-3′)	Size (bp)	References
Class 1 integrase	*Int* 1-F *Int* 1-R	AAGGATCGGGCCTTGATGTT CAGCGCATCAAGCGGTGAGC	471	Zarrilli et al. (40)
5’CS & 3’CS	5’CS 3’CS	GGCATCCAAGCAGCAAG AAGCAGACTTGACCTGA	721–741	Zarrilli et al. (40)
*aac*A1	*aac*(6′)-Ia	TAATTGCTGCATTCCGC	-	Lévesque et al. (41)
*aac*A4	*aac*(6′)-Ib	TGTGACGGAATCGTTGC	-	Lévesque et al. (41)
*bla*_PSE-1_	*bla*_PSE-1_ -F *bla*_PSE-1_ -R	GCAAGTAGGGCAGGCAATCA GAGCTAGATAGATGCTCACAA	422	Chuanchuen et al. (2009)
*bla*_TEM_	*bla*_TEM_-F *bla*_TEM_-R	ATCAGTTGGGTGCACGAGTG ACGCTCACCGGCTCCAGA	608	Chuanchuen et al. (2009)
*tet*A	*tet*A-F *tet*A-R	GCTGTCGGATCGTTTCGG CATTCCGAGCATGAGTGCC	658	Chuanchuen et al. (2009)
*tet*B	*te*tB-F *tet*B-R	CTGTCGCGGCTACGGTCAT CAGGTAAAGCGATCCCACC	615	Chuanchuen et al. (2009)
*cat*A	*cat*A-F *cat*A-R	CCAGACCGTTCAGCTGGATA CATCAGCACCTTGTCGCCT	454	Chuanchuen et al. (2009)
*cat*B	*cat*B-F *cat*B-R	CGGATTCAGCCTGACCACC ATACGCGGTCACCTTCCTG	461	Chuanchuen et al. (2009)
*cml*A	*cml*A-F *cml*A-B	TGGACCGCTATCGGACCG CGCAAGACACTTGGGCTGC	641	Chuanchuen et al. (2009)
*sul*1	*sul*1-F *su*l1-R	CGGACGCGAGGCCTGTATC GGGTGCGGACGTAGTCAGC	591	Chuanchuen et al. (2009)
*dfr*A1	*dfr*A1-F *dfr*A1-R	CAATGGCTGTTGGTTGGAC CCGGCTCGATGTCTATTGT	254	Chuanchuen et al. (2009)
*dfr*A10	*dfr*A10-F *dfr*A10-R	TCAAGGCAAATTACCTTGGC ATCTATTGGATCACCTACCC	432	Chuanchuen et al. (2009)
*dfr*A12	*dfr*A12-F *dfr*A12-R	TTCGCAGACTCACTGAGGG CGGTTGAGACAAGCTCGAAT	330	Chuanchuen et al. (2009)

The DNA template was prepared by selecting a pure colony on McConkey Agar, incubated at 37°C for 18–24 h. The colony was then transferred to 50 μL of TE buffer and boiled at 100°C for 5 min. The DNA fraction was then centrifuged at 11,000 rpm for 1 min, and the supernatant was collected for PCR testing. The PCR solution was prepared using 100 μL of Eppendorf® Master Mix (Eppendorf, Hamburg, Germany) containing 75 μL of sterile distilled water, 10 μL of 10x Tag buffer (100 mM Tris–HCL, pH 8.3), 50 μL MgCl_2_, 3 μL of 10 mM dNTPs, 1 μL of F-R primer (10 pmol/μL), 5 μL of DNA template, and Tag DNA polymerase (Apsalagon 5 U/μL). The first step of initial denaturation was set at 94°C for 5 min for 1 cycle, followed by DNA amplification in 3 steps for a total of 30 cycles: denaturation at 94°C for 30 s, annealing at 55°C for 30 s, and extension at 72°C for 30 s. The final step was an extension at 72°C for 5 min, 1 cycle. The PCR product (2 μL) was mixed with an equal volume of gel red (Biotium, California) and loaded for electrophoresis in 1% agarose gel (Axygen Biosciences, USA) at 100 V for 30 min. A ladder was used as the marker (Solis Biodyne, Estonia), and the results were interpreted on a UV-trans illuminator (Gel DocTM EZ Imager).

## Results

### Sample collection

**Figure 1:**
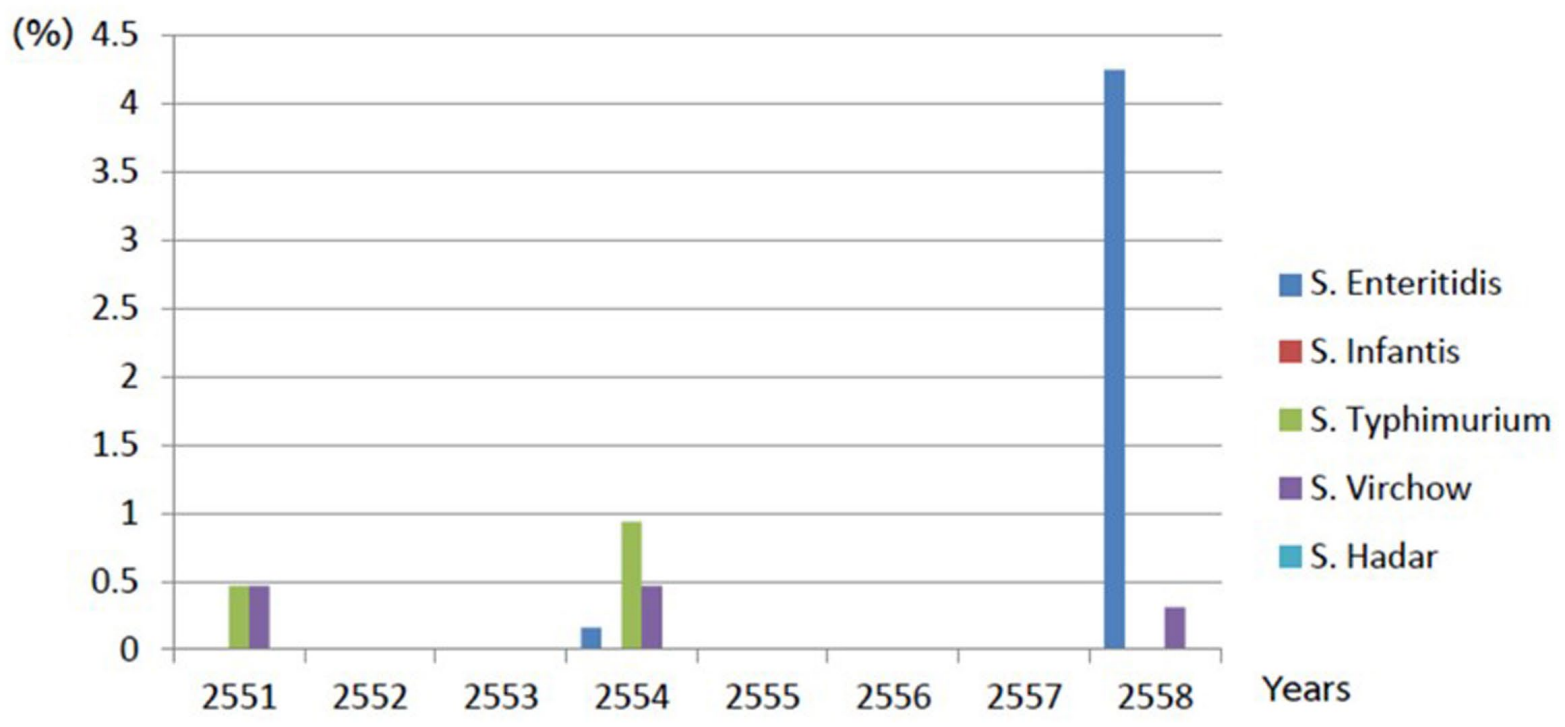
Demonstrates of *Salmonella* non-typhoidal found in livestock farm during 2008–2015.

This retrospective study gathered *Salmonella* spp. from livestock and their environments during 2008–2015. Among 636 samples, 86 samples were collected from swine farms, 39 from cleaning water within the farm, 24 from nursery piglets’ floor swabs, 18 from the soil,4 from drinking water (before and after chlorine treatment), 529 from duck farms and 21 samples were from the broiler farms. However, no *Salmonella* of non-host-specific serovars was detected during 2009–2010 and 2012–2014, as shown in Figure 1.

### Serovar identification

Among 45 non-host-specific *Salmonella* isolates, 9 were identified as *S.* Typhimurium, 28 as *S. enteritidis*, and 8 as *S*. Virchow. None isolates of *S. infantis* or *S.* Hadar were detected. In detail, it was indicated that *S.* Typhimurium was detected in 1.42% (9/636 samples: 3 environmental and 6 cloacal swabs), *S. enteritidis* in 4.40% (28/636 samples: 4 environmental and 24 cloacal swabs), and *S*. Virchow in 1.26% (8/636 samples: 8 cloacal swabs), as shown in Table 2.

**Table 2:** Demonstrate of *Salmonella* non-typhoidal isolated from livestock and its environment.

Livestock	Number	*S. enteritidis*	*S*. Hadar	*S*. Infantis	*S.* Typhimurium	*S*. Virchow
Swine farm samples	86					
Housing cleaning water (before/after)	39	−	−	−	+	−
Drinking water (before/after treatment	4	−	−	−	−	−
Soil sample	19	−	−	−	+	−
Nursery floor sample	24	+	−	−	−	−
Poultry farm samples	550					
Cloacal swab (duck)	529	+	−	−	+	+
Cloacal swab (broiler)	21	+	−	−	−	−
Total	636					

+; detected, −; non-detected.

### Drug resistance testing

**Figure 2:**
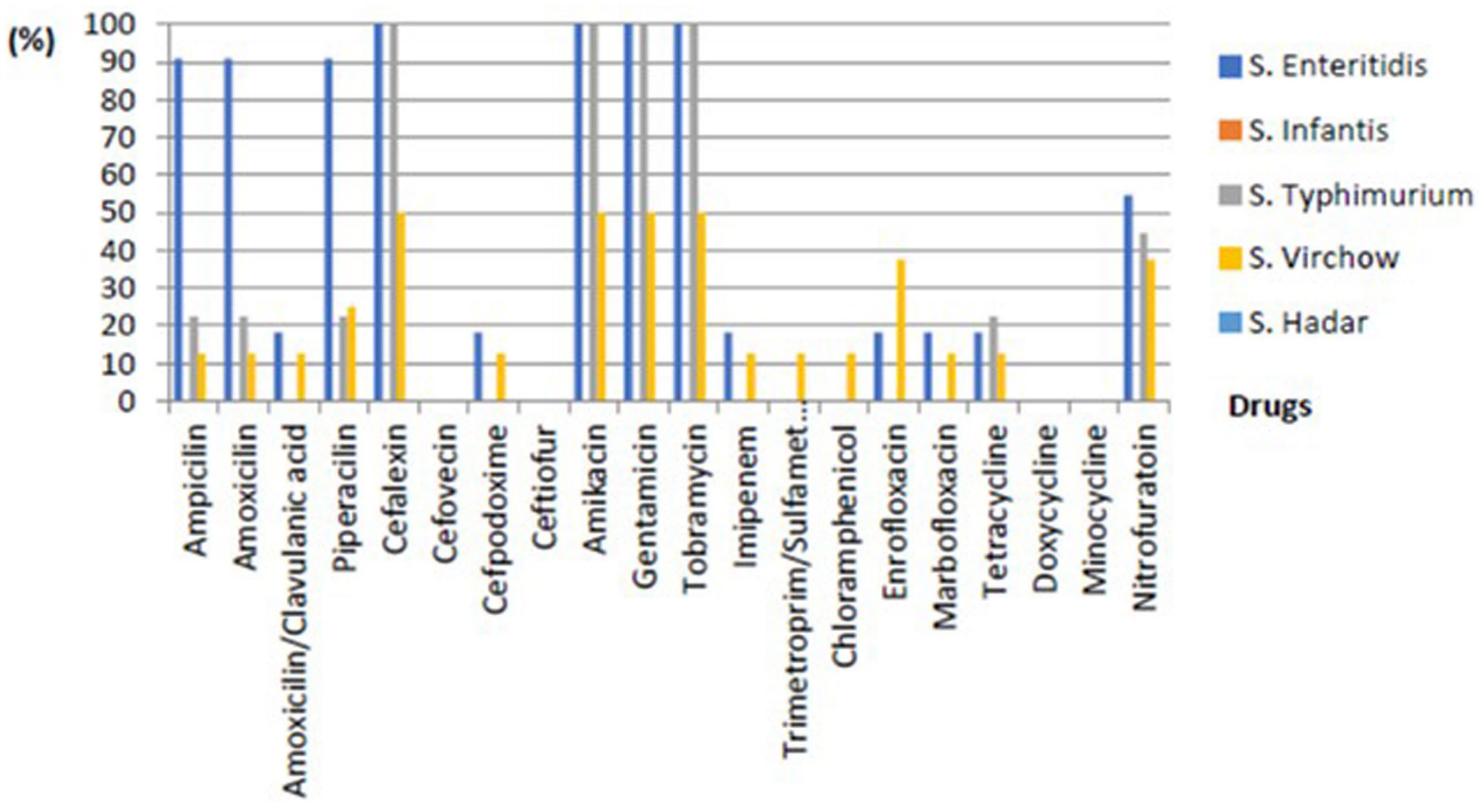
The percentage of phenotypic resistance of non-typhoidal *Salmonella* of each subtype from environmental and livestock samples.

Drug sensitivity testing was performed using the VITEK II system according to CLSI Vet03-S2 2014 standards. *S.* Typhimurium showed resistance rates of 22.22% towards Ampicillin, Amoxicillin, Piperacillin, and Tetracycline; 44.44% to Nitrofurantoin; and 100% to Cephalexin, Amikacin, Gentamicin, and Tobramycin. *S*. Virchow exhibited resistance rates of 12.50% towards Ampicillin, Amoxicillin, Amoxicillin-clavulanic acid, Imipenem, Sulfamethoxazole-trimethoprim, Chloramphenicol, Marbofloxacin, and Tetracycline; 25% for Piperacillin; 37.5% for Enrofloxacin and Nitrofurantoin; and 50% for Cefpodoxime, Amikacin, and Tobramycin. Lastly, *S. enteritidis* demonstrated resistance rates of 18.18% for Amoxicillin-clavulanic acid, Cefpodoxime, Imipenem, Enrofloxacin, Marbofloxacin, and Tetracycline; 54.55% for Nitrofurantoin; 90.91% for Ampicillin, Amoxicillin, and Piperacillin; and 100% for Cephalexin, Amikacin, Gentamicin, and Tobramycin and shown in Figure 2.

### Study of drug resistance gene cassette in integron by PCR

**Figure 3:**
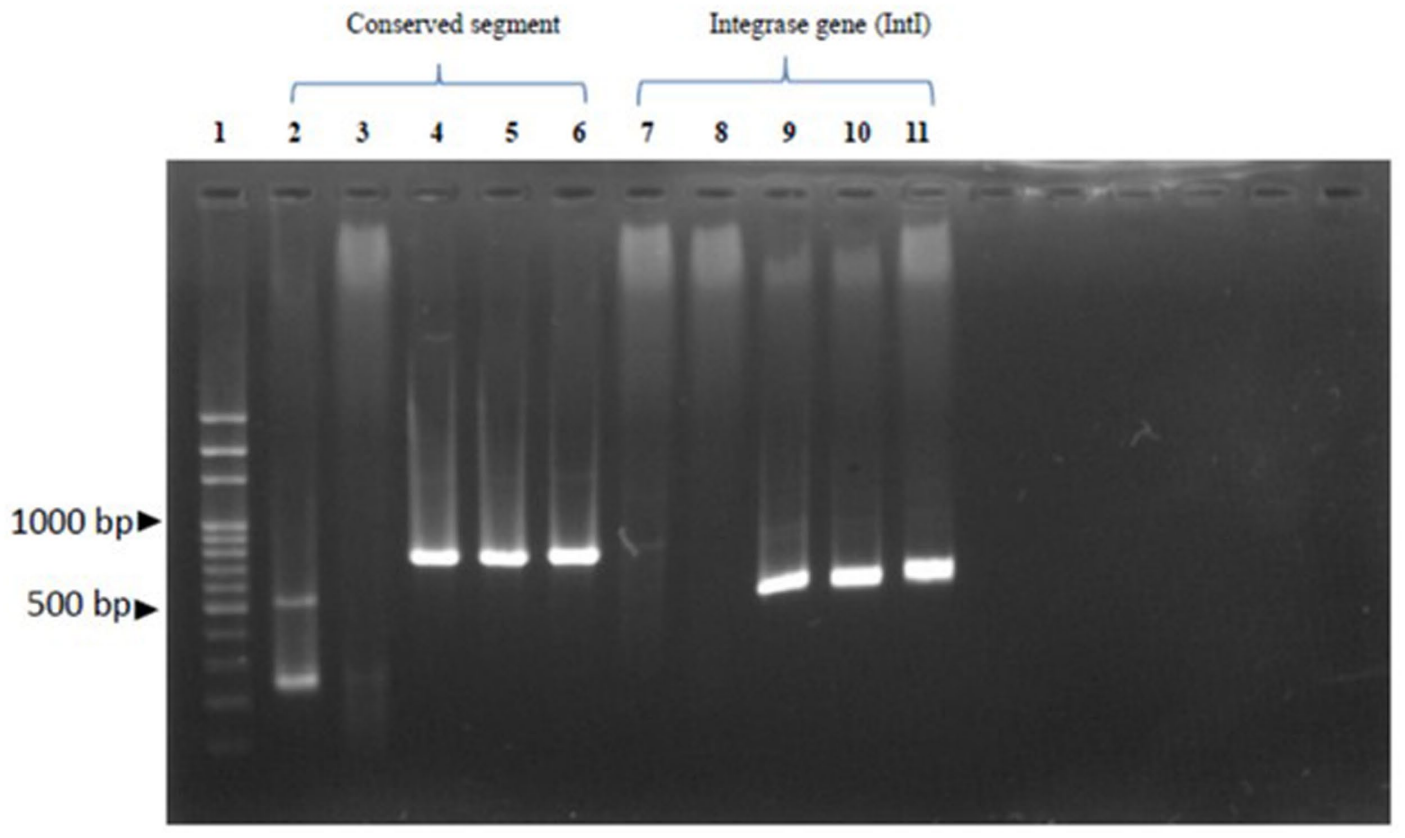
Demonstrates of the antibiotic resistance gene: Row 1 reveals 100 bp DNA Ladder Ready to load (Solis Biodyne, Tartu Estonia.); Row 2-6 reveals conserved segment (5’CS-3’CS) which row 2 and 3 were *S*. Typhimurium, row 4 and 5 were *S. Enteritidis* and row 6 was *S. Virchow*. Row 7-11 revealed of intergron class I by integrase gene (IntI) which row 7 and 8 were *S*. Typhimurium, row 9 and 10 were *S. Enteritidis* and row 11 was *S. Virchow*, respectively.

Gene cassette detection following MIC testing revealed resistance rates of 22.22% for *S.* Typhimurium, 18.18% for *S. enteritidis*, and 25% for *S*. Virchow, with an overall detection rate of 20.83%. Among the conserved segments found, the integrase gene was detected in 60% of cases, with 100% detection in both *S. enteritidis* and *S*. Virchow, but not detected at all in *S.* Typhimurium, as shown in Table 3 and Figure 3. According to Falagas and Karageorgopoulos (13), the concept of multidrug resistance indicates that bacteria resist at least three antibiotics. In this study, the aadA1 resistance gene, associated with Spectinomycin and Streptomycin resistance, was detected in 54.17% of isolates, with detection rates of 66.67% in *S.* Typhimurium, 8.33% in *S. enteritidis*, and 4.17% in *S*. Virchow. The *aad*A2 gene showed a resistance rate of 21.83%, with 22.22, 18.18, and 25%, respectively. The *aad*B gene against gentamicin was detected in 18.56% of cases, with 12.5, 18.18, and 25% detection in the same order. Using specific primers for the integron gene cassette on *Salmonella* conserved segments, they found of 100% *Int*I1 detected for *S. enteritidis* and *S*. Virchow but not for *S.* Typhimurium. Whereas, the study of *aac*(6′)-Ia resistance gene for Amikacin was detected in 100% of both *S. enteritidis* and *S*. Virchow, but none detected for the *aac*(6′)-Ib gene in these subtypes. The *bla*_PSE_-1 gene from the *β*-lactam group was not detected in all isolated *Salmonella*, but the *bla*_TEM_ gene was detected in 80% of cases, with 50, 100, and 100% detection for *S.* Typhimurium, *S. enteritidis,* and *S*. Virchow, respectively. The *tet*A gene for Tetracycline resistance was detected in 40% of cases, with 100% detection only for *S.* Typhimurium. The *tet*B gene showed an 80% detection rate, divided into 50, 100, and 100% in the same order. The *cat*A gene for chloramphenicol resistance was found in 80% of cases (50, 100, 100%), the *cat*B gene in 60% of cases (100, 100, 60%), and the *cml*A gene in 60% of cases (100% detection for *S. enteritidis* and *S*. Virchow). Lastly, the *sul*I-*drf*A1 resistance gene towards Sulfamethoxazole-trimethoprim was detected in 60% of cases (100% detection for *S. enteritidis* and *S*. Virchow), while the *dfr*A10 and *dfr*A12 genes were not detected in isolated *Salmonella* spp. as shown in Table 4.

**Table 3:** The resistance genes of *Salmonella* spp. of 3 subtypes isolate.

Gene	Subtype			
	Drug	Number (%)	Number (%)	Number (%)
		*S.* Typhimurium	*S. enteritidis*	*S.* Virchow
		(n = 9)	(n = 11)	(n = 4)
Conserved segment	–	2 (22.22)	18.18 (2.0)	25 (1.0)
*aad*A1	Spectinomycin/Streptomycin	6 (66.67)	6 (54.54)	25 (1.0)
*aad*A2	Spectinomycin/Streptomycin	2 (22.22)	2 (18.18)	25 (1.0)
*aad*B	Aminoglycoside	0 (0)	2 (18.18)	25 (1.0)

Gene	Subtype			
	Drug	Number (%)	Number (%)	Number (%)
		*S.* Typhimurium	*S. enteritidis*	*S*. Virchow
		(n = 2)	(n = 2)	(n = 1)
*IntI*1	Integron	0 (0)	2 (100)	1 (100)
*aac*(6′)-Ia	Amikacin	0 (0)	2 (100)	1 (100)
*aac*(6′)-Ib	Amikacin	0 (0)	0 (0)	0 (0)
*bla*_PSE-1_	β-lactam	0 (0)	0 (0)	0 (0)
*bla*_TEM_	β-lactam	1 (50)	2 (100)	1 (100)
*Tet*A	Tetracycline	2 (100)	0 (0)	0 (0)
*Tet*B	Tetracycline	1 (50)	2 (100)	1 (100)
*Cat*A	Chloramphenicol	1 (50)	2 (100)	1 (100)
*Cat*B	Chloramphenicol	2 (100)	0 (0)	1 (100)
*Cml*A	Chloramphenicol	0 (0)	2 (100)	1 (100)
*Sul*	Sulfamethoxazole	0 (0)	2 (100)	1 (100)
*dfr*A1	Trimethoprim	0 (0)	2 (100)	1 (100)
*dfr*A10	Trimethoprim	0 (0)	0 (0)	0 (0)
*dfr*A12	Trimethoprim	0 (0)	0 (0)	0 (0)

**Table 4:** Demonstration of phenotype and drug resistance genes integron cassettes.

Subtype	Phenotype	Genotype	*Int*I1	5′-CS and 3′-CS
*S.* Typhimurium	Am Ax Cep An Gm Te	*aad*A1 *tet*A tetB *cat*B	−	+
*S.* Typhimurium	Cep An Gm	*aad*A2 *bla*_PSE-1_ *tet*A catA *ca*tB	−	+
*S. enteritidis*	Am Ax Cep An Gm Qu Te	*aad*B *aad*A1 *aad*A2 *aac*(6′)-Ia	+	+
*S. enteritidis*	Am Ax Cep An Gm Qu Te	*bla*_TEM_ *tetB cat*A *cml*A *sul*I *dfr*A1	+	+
*S.* Virchow	Am Ax Cep An Gm Sul-Tri Qu Te	*aad*B *aad*A1 *aad*A2 *aac*(6′)-Ia	+	+
		*bla*_TEM_ *tet*B *cat*A *cat*B *cml*A *sul*I *dfr*A1	+	+

Am, Amoxycillin; Ax, Amoxycillin; Cep, Cephalexin; An, Ampicillin; Gm, Gentamicin; Te, Tetracyclin; Qu, Quinolone; Sul-Tri, Sulfamethoxazole-trimethoprim.

## Discussion

According to former reports between 2003 to 2006 in Thailand, 80% of *S.* Typhimurium isolated from environmental samples showed antibiotic resistance, especially in the pig farm environments (14). In this study, Non-Typhoidal *Salmonella* spp. (NTS) collected from pig farm environments were detected at the rate of 33.33% (3 out of 9 samples), they all were collected from cleaning water and contaminated soil samples. In northern Thailand, *S.* Typhimurium and *S. enteritidis* were found in 18.44 and 1.78% of samples, respectively. However, 28% isolation rate from retail chicken meat was also reported (10, 15). *S. enteritidis* is commonly found on pig farm floors, which similar to the others finding of 3.12% in pig farm environments and 6.6% from chicken feces sample (15, 16). Interestingly, this finding was different from the current studied of *Salmonella Enterica* isolated from animal feedstuffs in year 2017, they reported that the most serotypes found were *S*. Rissen, *S*. Mbandaka and *S.* Livingstone, respectively (17).

There was also a report of finding 66% of *S.* Typhimurium in pigs and farm environments in Spain, which is a significant problem for both *S.* Typhimurium and *S. enteritidis* in America. Additionally, *S. enteritidis* and *S.* Typhimurium have been isolated from broiler farms and their environments in Thailand (12), which correlates well with the 7.81% detection rate for both serotypes in broiler farms in Algeria (18) and the presence of *S*. Virchow in broiler farms in China (19).

### Antibiotic resistance study

This study demonstrated antibiotic resistance in NTS, specifically *S.* Typhimurium, *S. enteritidis*, and *S*. Virchow. Resistance to one or two antibiotics was observed in 37.5% of cases, while 62.5% exhibited MDR. Among the MDR isolates, resistance to more than three drugs were found in 38.4% (5/13) of duck samples, 83% (5/6) of pig samples, and 100% (5/5) of broiler samples. They were resistant to beta-lactam, Cephalosporins, and Aminoglycoside antibiotics.

Comparing these findings with previous reports on *Salmonella* spp. isolated from broiler and pig feces from 2003 to 2005 in central Thailand, it was found that the bacteria were 100% sensitive to Gentamicin and Ciprofloxacin (9). However, another study reported bacterial resistance in broilers, especially *S. enteritidis* and *S*. Virchow, with 100% resistance to Nalidixic acid and only 16.7% resistance to Kanamycin. Studies in Vietnam and Thailand demonstrated of antibiotic resistance rates of 28 and 59%, respectively, particularly for ampicillin and tetracycline (20). During 2004–2007, there were reports of high antibiotic resistance for Nalidixic acid, Ciprofloxacin, and Ampicillin (21), with an increasing trend in resistance to Cephalosporins and Aminoglycosides. In addition, the study of *S. enterica* isolated from animal feedstuffs in Thailand, of which they found MDR in those isolates, therefore, the commercial feeds and raw material involved people should be monitored (17).

### Resistance gene detection

The MIC testing for the three serotypes revealed resistance to Aminoglycosides, with the detection of *aad*A1*, aad*A2, and *aad*B genes. This finding is consistent with reports of the *aad*A1 gene in *Salmonella* spp. isolated from pigs and chickens (22, 23), as well as similar findings in pig and chicken farms in Thailand (24). In this study, *S.* Typhimurium isolated from swine environments showed resistance to beta-lactam, Cephalosporin, Aminoglycoside, and Tetracycline antibiotics. The resistance genes *aad*A1, *tet*A, *tet*B, and *cat*B were well-correlated with findings abroad and in Chloramphenicol resistance (12, 23).

There have been reports of the detection of *aad*A2, *bla*PSE-1, *tet*A, *cat*A, and *cat*B in gene cassettes from *S.* Typhimurium, indicating MDR (25). The detection of multiple resistance genes in *S.* Typhimurium suggests the potential for future MDR development. This study found that *S. enteritidis* was resistant to Aminoglycosides, Cephalosporins, beta-lactams, Quinolones, and Tetracycline. The detected genes included *aad*B, *aad*A1, *aad*A2, *aac*(6′)-la, *bla*TEM, *tet*B, *cat*A, *cml*A, *sul*I, and *dfr*A1, with genotypic patterns similar to *S. enteritidis* isolated from broilers in Romania (22), indicating MDR. Moreover, *S*. Virchow in this study showed resistance to Aminoglycosides, Cephalosporins, beta-lactams, Quinolones, Tetracycline, and Sulfamethoxazole/trimethoprim. The phenotypic pattern included *aad*B, *aad*A1, *aac*(6′)-la, *bla*TEM, *tet*B, *cat*A, *cat*B, *cml*A, *sul*I, and *dfr*A1, which were consistent with gene cassettes *drf*A1,2-*ort*f-*aad*A2 in *S.* Typhimurium showing MDR (26). The gene patterns detected in *S*. Virchow were similar to those in *S. enteritidis*, *S.* Typhimurium, and indicating potential MDR (27).

### Class I integron and MDR

Chuanchuen et al. (12) and Partridge et al. (28) reported that MDR in *Salmonella* spp. is caused by abundant gene cassettes in class I integron (*Int*I), which can be mobile genetic elements (MGEs) in gram-negative bacteria (25, 29). Since MGEs composed of various elements such as integrons, transposons and plasmids which were considered to responsible for the Horizontal gene transfer (HGT). HGT is crucial for the propagation of increased of antibiotic resistance (30, 31). The gene cassettes in *S. enteritidis* isolated from chickens in China indicated MDR, with a resistance rate of 65% by MIC testing (CLSI Vet03-S2 2014 NCCLS standard) and a 33.3% detection rate for gene cassettes. Two different gene cassette sizes were detected, distinguishing *S.* Typhimurium from *S. enteritidis* and *S*. *Virchow*, which had identical cassette sizes. The gene cassette size difference indicates structural differences in the genes (FEMS Microbial Rev., 2009). In this study, IntI gene cassettes were detected in 60% (3/5) of *S.* Typhimurium and 100% (2/2) and 100% (1/1) for *S. enteritidis* and *S*. Virchow, respectively, whereas the *Int*I may induce MDR in *S. enteritidis* and *S*. Virchow in broilers. Since, class 1 integrons is one of the major integrons classes, which normally associated with the HGT of antibiotic resistance and it could be existed in environmental sample with sequence diversity (32, 33). The antimicrobial resistance in broiler farms was higher than in the native chickens, attributed to high use of antibiotics in the broiler industry. Therefore, careful recognition of antibiotic use in farms is necessary attributed to the increasing trend of MDR in *Salmonella* spp. especially in developed and developing countries (34). Even after the cessation of antibiotic use, the residues and persistence of the resistance genes in environment may increase the likelihood of MDR in *Salmonella* which could be entering the food chains (35, 36).

There was report indicated the similar MDR serotype of *Salmonella* spp. and *E. coli* could be detected in farm workers, infected pigs and their environment in Thailand (37, 38). The presence of MDR circulation in the food web poses significant to public health and medical concerns, since the incidents of antibiotic resistance in NTS have been reported in America (39), Thailand and Vietnam (20). Once an outbreak occurs, prevention and control measures become challenging and serious concern for global public health.

## Conclusion

This study identified an increasing trend in antibiotic resistance, particularly MDR, among *Salmonella* isolates from Nakhon Pathom and Suphan Buri provinces, especially for the three serotypes of examined. The observed resistance patterns are likely linked to the continued use of antibiotics in livestock production systems. Despite laws regulating the antibiotics and control strategies enforcing of reasonable use, the long-term misuse of antibiotics in livestock still persists. This misuse leads to environmental contamination and the spread of resistance genes into the bacteria, particularly *Salmonella* as known as a major foodborne pathogen in food safety.

Therefore, prevention and control strategies are essential to delaying antibiotic resistance in bacteria. The determination of class I integron genes and gene cassettes in *Salmonella* spp. serves as a valuable tool for understanding resistance of the bacteria and monitoring MDR in the livestock. This study framework can be applied for further explore the relationship between IntI and resistance gene cassettes of *Salmonella* isolates from livestock and their environments. These findings are critical for developing the predictive tools and effective MDR prevention and control strategies for livestock production industry which become one of a major industry in Thailand.

## Data Availability

The raw data supporting the conclusions of this article will be made available by the authors, without undue reservation.
